# A *Staphylococcus aureus* Small RNA Is Required for Bacterial Virulence and Regulates the Expression of an Immune-Evasion Molecule

**DOI:** 10.1371/journal.ppat.1000927

**Published:** 2010-06-03

**Authors:** Svetlana Chabelskaya, Olivier Gaillot, Brice Felden

**Affiliations:** Université de Rennes I, Inserm U835, Upres EA2311, Biochimie Pharmaceutique, Rennes, France; Institut Pasteur, France

## Abstract

*Staphylococcus aureus*, a pathogen responsible for hospital and community-acquired infections, expresses many virulence factors under the control of numerous regulatory systems. Here we show that one of the small pathogenicity island RNAs, named SprD, contributes significantly to causing disease in an animal model of infection. We have identified one of the targets of SprD and our *in vivo* data demonstrate that SprD negatively regulates the expression of the Sbi immune-evasion molecule, impairing both the adaptive and innate host immune responses. SprD interacts with the 5′ part of the *sbi* mRNA and structural mapping of SprD, its mRNA target, and the ‘SprD-mRNA’ duplex, in combination with mutational analysis, reveals the molecular details of the regulation. It demonstrates that the accessible SprD central region interacts with the *sbi* mRNA translational start site. We show by toeprint experiments that SprD prevents translation initiation of *sbi* mRNA by an antisense mechanism. SprD is a small regulatory RNA required for *S. aureus* pathogenicity with an identified function, although the mechanism of virulence control by the RNA is yet to be elucidated.

## Author Summary

Bacteria possess numerous and diverse means of gene regulation using RNA molecules, including small RNAs (sRNAs). Here we show that one sRNA is essential for a major human bacterial pathogen, *Staphylococcus aureus*, to cause a disease in an animal model of infection. Our study provides evidence that this RNA regulates the expression of an immune evasion molecule secreted by the bacterium to impair the host immune responses, and we have solved the mechanism of the RNA-based regulation at molecular level. So far, the mechanism of bacterial virulence controlled by SprD is unrevealed, but that small RNA has a huge impact in the course of a bacterial infection. It implies possible new strategies in fighting against that major human and animal bacterial pathogen in preventing the expression of this regulatory RNA.

## Introduction

*Staphylococcus aureus* is a member of the commensal flora that can be an opportunistic pathogen and a cause of nosocomial and community-acquired infections [1]. With the widespread use of antimicrobials, the incidence and spread of highly antibiotic-resistant *S. aureus* strains have increased rapidly in recent years and constitute a clinical and epidemiological challenge in hospitals all over the world. In order to survive and to establish an infection, *S. aureus* inhibits the attack of the host immune system, utilizing diverse escape mechanisms [2]. The staphylococcal protein A (SpA) recognizes the Fc domain of immunoglobulins which results in inverted tagging and blocking the C1q and Fcγ receptor binding sites [3]. *S. aureus* IgG binding protein (Sbi) is another immunoglobulin-binding protein expressed by *S. aureus* [4]. Sbi acts also as a complement inhibitor and forms a tripartite complex with host complement factors H and C3b [5].

*S. aureus* modulates the expression of virulence genes in response to environmental changes thanks to global regulatory elements. They are either two-component regulatory systems as the *agr* (*a*ccessory *g*ene *r*egulator) regulon which is a sensor of the population density [6], or transcription factors as the SarA family of DNA binding-proteins [7]. These pathways allow the expression of virulence factor regulation during host colonization and dissemination. In addition to protein-mediated regulations, ribonucleic acids also possess regulatory functions in many bacterial pathogens [8]. Until now, RNAIII is the only *S. aureus* regulatory RNA with a demonstrated function. It is the effector of the global *agr* regulon that controls the synthesis of several virulence factors [9], [10]. RNAIII regulates the expression of numerous mRNA targets at the translational and/or transcriptional levels [11] and also acts as an mRNA, containing a small ORF encoding the delta-hemolysin.

Additional regulatory RNAs are expressed by *S. aureus* [12]–[14]. Their expression profiles vary among clinical strains and many of them, called **Spr** for ‘**s**mall **p**athogenicity island **r**NAs’, are expressed from genomic pathogenicity islands containing virulence and antibiotic resistance genes. Their functions are so far unknown. This study was aimed at elucidating the role of one of them, SprD. The Sbi immune evasion protein was identified as a molecular target of SprD. We show that SprD interacts with the *sbi* mRNA by an antisense mechanism, occluding the Shine-Dalgarno (SD) sequence and the initiation codon. Moreover, we show that a small regulatory RNA SprD has a major implication during the intravenous (i.v.) infection of mice by a *S. aureus* clinical strain.

## Results

### SprD expression profile during growth

**Figure 1:**
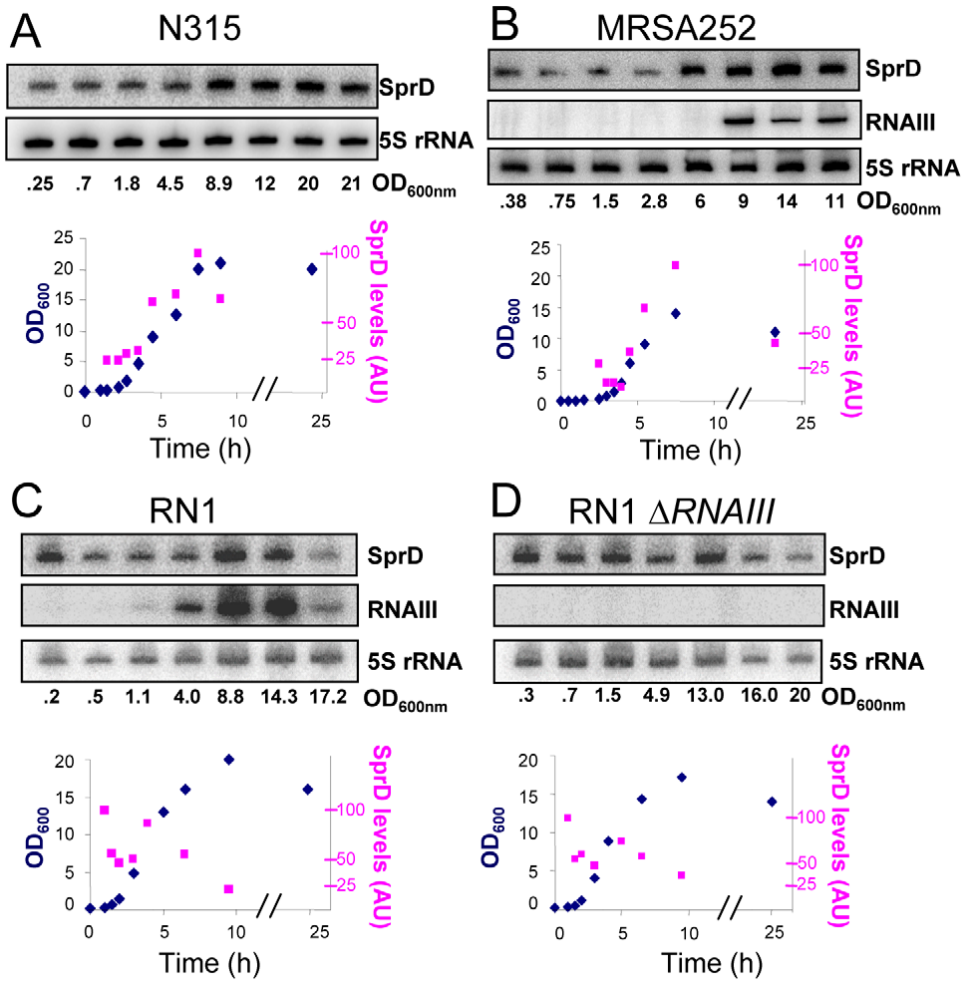
SprD RNA expression profiles in several *S. aureus* strains. The expression of SprD during a 24-hour growth of *S. aureus* N315 (**A**), MRSA252 (**B**), RN1 (**C**) and RN1 Δ*RNAIII* (**D**) strains by Northern blots using labeled DNA probes for SprD and for the RNAIII. As loading controls, the blots were also probed for 5S rRNAs. The growth curves of N315 (**A**), MRSA252 (**B**), RN1(**C**) and RN1 Δ*RNAIII* (**D**) strains are presented, with the quantification of the SprD levels in the four strains relative to the amount of 5S rRNAs from the same RNA extraction, the maximum value of SprD expression for each strain was normalized to 100. (AU, arbitrary units).

The expression of SprD was monitored during the growth of N315 (*agr−*) [15], [16] and MRSA252 (*agr+*) [17], two *S. aureus* clinical isolates. SprD is already expressed during the early exponential (E) phase (**Figure 1**), in contrast to RNAIII that is transcribed at late exponential and stationary (S) growth phases. SprD expression increases during the E phase, up to the end of the E phase for N315 and MRSA252 strains. To evaluate the implications of the RNAIII in SprD expression during growth, an RNAIII deletion mutant (Δ*RNAIII*) was constructed in strain RN1 (*agr*+) [18]. In contrast to N315 and MRSA252, the SprD expression levels remain almost identical during cell growth in strain RN1 (Figure 1C–D). Also, during growth SprD expression is similar in RN1wt and in RN1-*ΔRNAIII* isogenic strains (**Figure 1C–D**), suggesting that the expression pattern of SprD is not influenced by the RNAIII. Therefore, the expression of SprD is independent of the presence of absence of RNAIII.

### SprD regulates the expression of an immune-evasion protein at translational level

**Figure 2:**
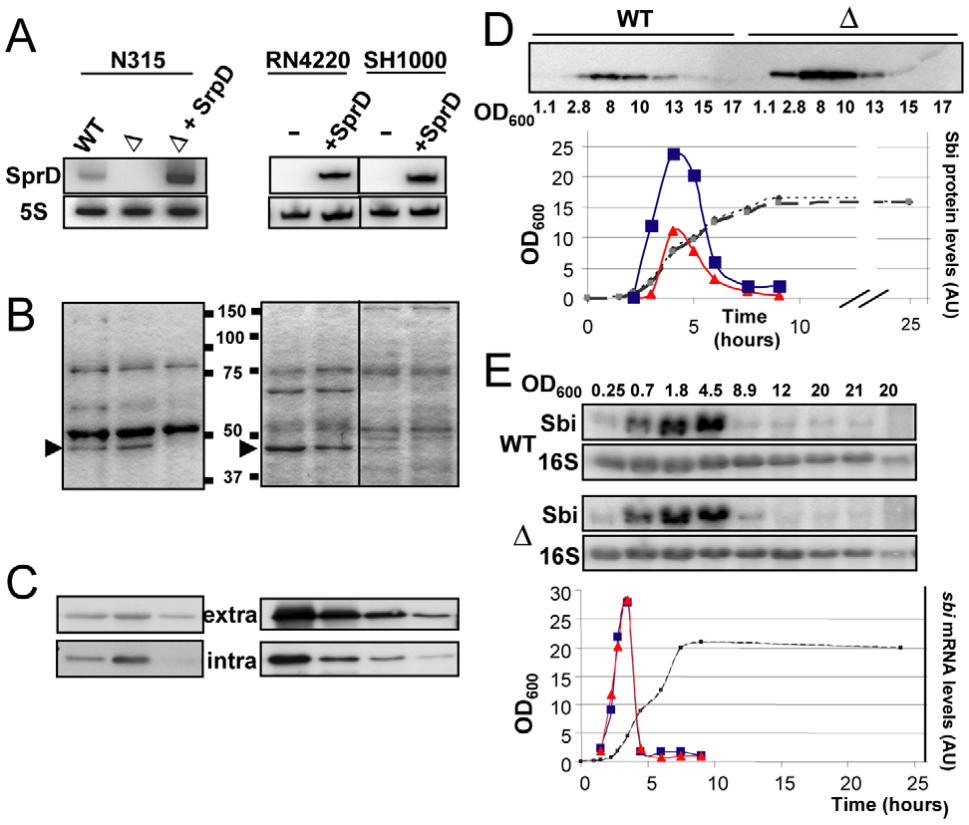
The SprD regulates expression of the Sbi protein at translational level *in vivo*. (**A**) Northern blot analysis of the SprD expression at the E phase (OD_600nm_: 2) in wild-type N315 (WT), N315 isogenic Δ*sprD* mutant (Δ),Δ*sprD* transformed by pCN38Ω*sprD* (complemented strain ‘ΔSprD’) and also in strains RN4220 and SH1000 transformed by pCN38ΩsprD. (**B**) Coomassie staining of SDS–PAGE of the exoproteins in N315, RN4220 and SH1000 strains expressing, or not, SprD at the E phase (OD_600nm_: 2). The arrows point to the reduced levels of a protein when SprD is expressed. (**C**) Immunoblot analysis with anti-Sbi antibodies of extra- and intracellular proteins in the three *S. aureus* strains at the E phase (OD_600nm_: 2). (**D**) Monitoring the expression of the Sbi protein during *S. aureus* growth in strains N315 (WT) and Δ*sprD* (Δ) by immunoblots with anti-Sbi antibodies separated on the same gel. The graph shows the quantification of the Sbi protein levels in both strains relative to the total protein amount (**Figure S5**). The blue squares represent the Δ*sprD* and the red triangles represent WT. The superimposable growth curves of the two strains are represented as the dashed lines. (**E**) Northern blot analysis of the *sbi* mRNA in wild-type N315 (WT) and Δ*sprD* mutant (Δ) during bacterial growth. 16S rRNAs are loading controls. The graph shows the quantification of the *sbi* mRNA levels in both strains relative to 16S rRNA and the colours correspond to panel D. We have measured the *sbi* mRNA half-life in WT SH1000 strain using rifampicin treatment, which is about 1 min (data not shown).

*SprD* is expressed from the genome of a converting phage containing virulence factors [12]. In most *S. aureus* strains, *sprD* is situated in-between *scn* and *chp*, within the 8 kb innate immune evasion cluster (IEC) that contains the genes for modulation of the early immune response. Such a genomic localization, as well as its growth phase dependent expression, suggest that this RNA may regulate the expression of virulence factor(s). In order to identify the target(s) of SprD, we analyzed whether SprD modifies the expression of extracellular proteins that contain many virulence factors. For this purpose, a *sprD* deletion mutant (Δ*sprD*) was constructed. We determined *sprD* 5′-end by RACE (rapid amplification of cDNA ends) at position C2007178 from the *S. aureus* N315 sequence [16]. Based on (*i*) 5′-end mapping, (*ii*) transcript size derived from Northern blot analysis [12] and (*iii*) transcription terminator prediction (**Figure S1**), *sprD* 3′-end was assigned at position G_2007037_, implying that SprD has 142 nts. In *S. aureus* N315, the *sprD* gene was substituted for an erythromycin resistance cassette by homologous recombination, abolishing the SprD expression (**Figure 2A**). Complementation of Δ*sprD* was achieved with a pCN38ΩsprD plasmid expressing SprD from its endogenous promoter (strain Δ*sprD*+SprD). In *S. aureus* strains RN4220 [19] and SH1000 [20] naturally lacking the *sprD* gene (**Figure 2A**), SprD was expressed with the pCN38Ω*sprD* plasmid (**Figure 2A**). In all three strains, levels of a ∼45kD protein decrease in the presence of SprD (**Figure 2B**). In the RN4220 strain, proteins from that band were eluted, a tryptic digest was prepared and the fragments analyzed by MALDI-TOF mass spectrometry. Twenty-five peptides were identified, all matching the sequence of the Sbi protein (**Table S1**). A confirmation of the decrease of the Sbi levels by SprD within the extracellular proteins was obtained by monitoring the Sbi protein by Western blots (**Figure 2C**). Interestingly, the SprD-dependent downregulation of the Sbi protein was also observable within the intracellular proteins (**Figure 2C**), indicating that the regulation does not affect Sbi protein export but the overall Sbi protein expression levels. Complementation of Δ*sprD* with the pCN38Ω*sprD* vector reduces the Sbi protein levels *in vivo* (**Figure 2**, panels **B** and **C**), demonstrating that SprD by itself regulates the expression of Sbi protein. These results were also obtained in strain RN1 and its isogenic Δ*sprD* mutant (data not shown).

Wild-type N315 and Δ*sprD* strains growth curves are superimposable in rich broth (**Figure 2D**), demonstrating that the SprD does not influence *S. aureus* proliferation. The complemented strain leads to lower Sbi protein levels compared to the wild-type N315 strain (**Figure 2**, panels **B** and **C**) because the expression of SprD from pCN38Ω*sprD* is higher than its endogenous expression levels in wild-type N315 strain (**Figure 2A**). In the N315 strain, the highest expression of the Sbi protein during growth is at mid-exponential phase and goes to zero at early stationary phase and beyond (**Figure 2D**). In its isogenic Δ*sprD* mutant, the Sbi protein levels are higher during growth but the expression profile remains similar (**Figure 2D**). Taken together, these data establish a functional link between the Sbi protein levels and the expression of SprD demonstrating that, in different *S. aureus* genetic backgrounds, SprD represses Sbi expression *in vivo*.

To test whether the regulation of Sbi by SprD is at transcriptional and/or at translational level(s), the *sbi* mRNA levels were monitored by Northern blots in wt N315 and Δ*sprD* strains. The *sbi* mRNA expression profiles are similar in both strains (**Figure 2E**), with a gradual increase of the mRNA expression up to the early-exponential phase and a sharp decrease to basal levels later on. When the *sbi* mRNA strongly decreases at the stationary phase, SprD is not more expressed, indicating that the Sbi repression at the S phase is ‘SprD-independent’. Also, the *sbi* mRNA expression profile does not follow the protein synthesis pattern, probably meaning that the Sbi protein is stable and accumulates during growth. Therefore, in strain N315, the expression of the Sbi protein is dictated by its transcription profile during bacterial growth. In addition, SprD does not modify the steady state level of the *sbi* mRNA. Taken together, these results show that SprD downregulates Sbi expression at translational level.

### SprD interacts with the *sbi* mRNA by an antisense mechanism

**Figure 3:**
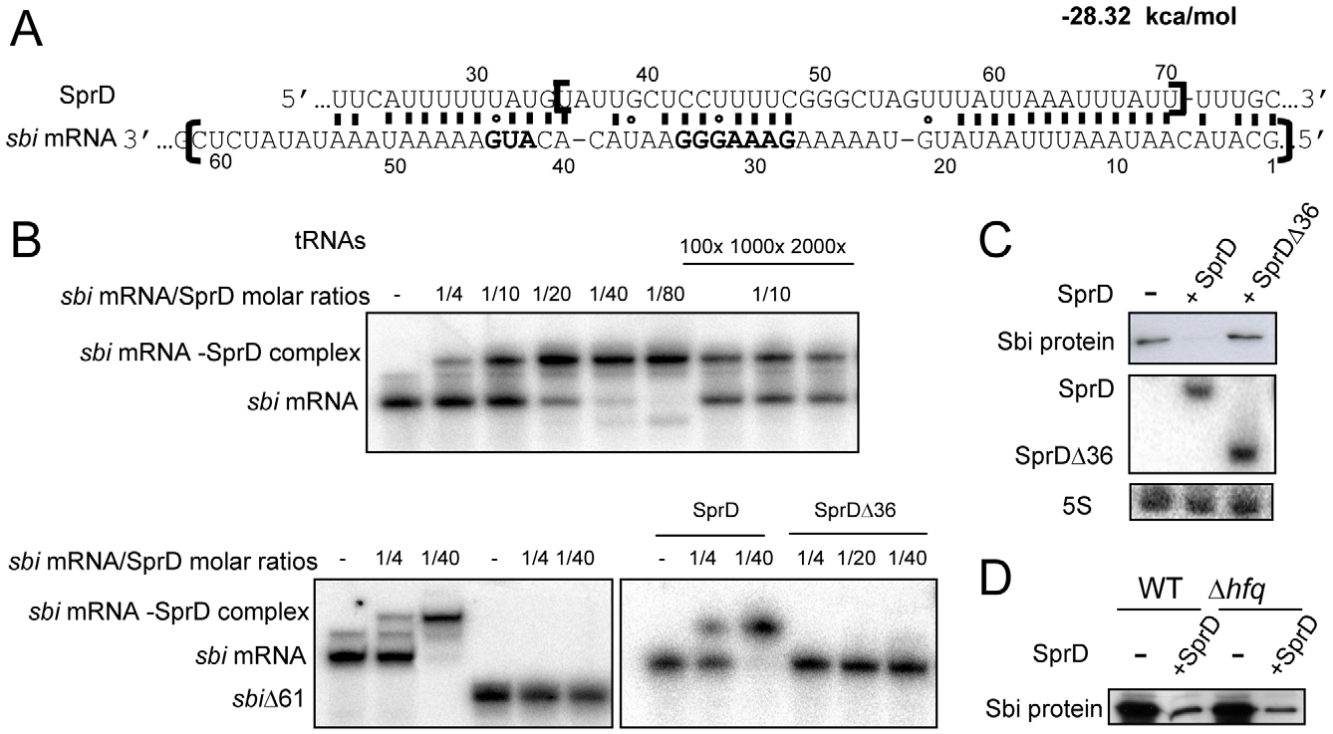
The regulation of Sbi by SprD involves a direct interaction between SprD and the *sbi* mRNA. (**A**) *In silico* prediction of an interaction between SprD and the *sbi* mRNA. The free energy of the SprD-*sbi* mRNA pairing is provided. The nucleotides bordered by two brackets were deleted in SprDΔ36 and in *sbi*Δ61. In the *sbi* mRNA sequence, the grey nucleotides are the putative SD (5′-GAAAGGG-3′) and the start codon. (**B**) Complex formation between SprD and the *sbi* mRNA. Native gel retardation assays of purified labeled *sbi* mRNAs (the *sbi* mRNA contains 179 nts at the mRNA 5′-end and *sbi*Δ61 contains 118 nts) with increasing amounts of either unlabeled SprD, mutant SprD lacking nts 35–70 (SprDΔ36) or of a 100 to 2000-fold excess of unlabeled *yeast* total tRNAs. (**C**) Monitoring *in vivo* the expression levels of the Sbi protein in strain N315 Δ*sprD* (−) complemented by either pCN38Ω*sprD* (+SprD), or by pCN38Ω*sprDΔ36* (+SprDΔ36) at E phase. Bottom panel: Northern blot analysis of SprDΔ36 and SprD RNAs, 5S rRNAs are the loading controls. (**D**) Monitoring the expression levels of the Sbi protein in the RN4220 WT and RN4220 Δ*hfq* isogenic strains, in the presence and absence of the SprD, by immunoblots with anti-Sbi antibodies.

We focused our next investigations on Sbi to elucidate the mechanism of its regulation by SprD. A substantial fraction of bacterial regulatory RNAs for which a function was identified interacts with target mRNAs to regulate gene expression [21]. Putative interactions between SprD and the 5′-portion of *sbi* mRNA were detected *in silico* (**Figure 3A**). We first determined the *sbi* mRNA transcriptional start site by RACE at position G_2476039_ from the N315 genomic sequence [16]. Therefore, *sbi* mRNA 5′-end is located 41 nts upstream of the AUG initiation codon (**Figure 3A**). Duplex formation between SprD and a 179 nts-long *sbi* mRNA fragment containing its 5′ UTR sequence followed by 46 codons was analyzed by gel retardation assays. A ‘SprD-*sbi* mRNA’ duplex was detected at a 1∶4 molar ratio and nearly all *sbi* mRNA was in complex with SprD at a 1∶20 molar ratio (**Figure 3B**). The binding is specific since a 100- to 2,000-fold molar excess of total tRNAs do not displace the *sbi* mRNA from a preformed ‘SprD-*sbi* mRNA’ complex. A *sbi* mRNA deletion mutant lacking 61 nts at its 5′-end (*sbi*Δ61, **Figure 3A**, brackets), predicted to be part of the interaction, does not bind SprD (**Figure 3B**), demonstrating that these nucleotides are required to interact with SprD. Reciprocally, the deletion of 36 nucleotides (U35 to U70) from SprD (SprDΔ36, **Figure 3A**, brackets) abolishes complex formation (**Figure 3B**), showing that these nucleotides are also required for the ‘SprD-*sbi* mRNA’ interaction.

To provide a direct evidence *in vivo* of the interaction between SprD and the *sbi* mRNA, we have expressed the SprDΔ36 RNA in the Δ*sprD* strain. Western blots indicate that SprDΔ36 RNA is unable to dowregulate the Sbi protein levels *in vivo*, in contrast to full-length SprD (**Figure 3C**). Northern blot indicates that the SprDΔ36 mutant RNA is expressed at similar levels than SprD wt, demonstrating that the absence of Sbi downregulation by the SprDΔ36 mutant RNA is not due to its instability *in vivo*. Therefore, this result is a strong evidence of a direct interaction between SprD and the *sbi* mRNA *in vivo*, as illustrated in **Figure 3A**. The interaction between the *sbi* mRNA and SprD forms *in vitro* without the contribution of a helper molecule (**Figure 3B**), as the Sm-like Hfq protein. To test the contribution of the Hfq protein *in vivo*, we have monitored the SprD-mediated regulation of Sbi in an *hfq* deletion strain versus an isogenic wild-type strain. As shown in **Figure 3D**, the *in vivo* regulation of Sbi expression by SprD takes place independently of the presence or absence of Hfq. These results demonstrate that SprD forms a stable complex with the *sbi* mRNA *in vitro* and *in vivo*, as well as deletions altering the complementarities between the two RNAs impair complex formation.

### The ribosome binding site of the *sbi* mRNA is sequestered by SprD

**Figure 4:**
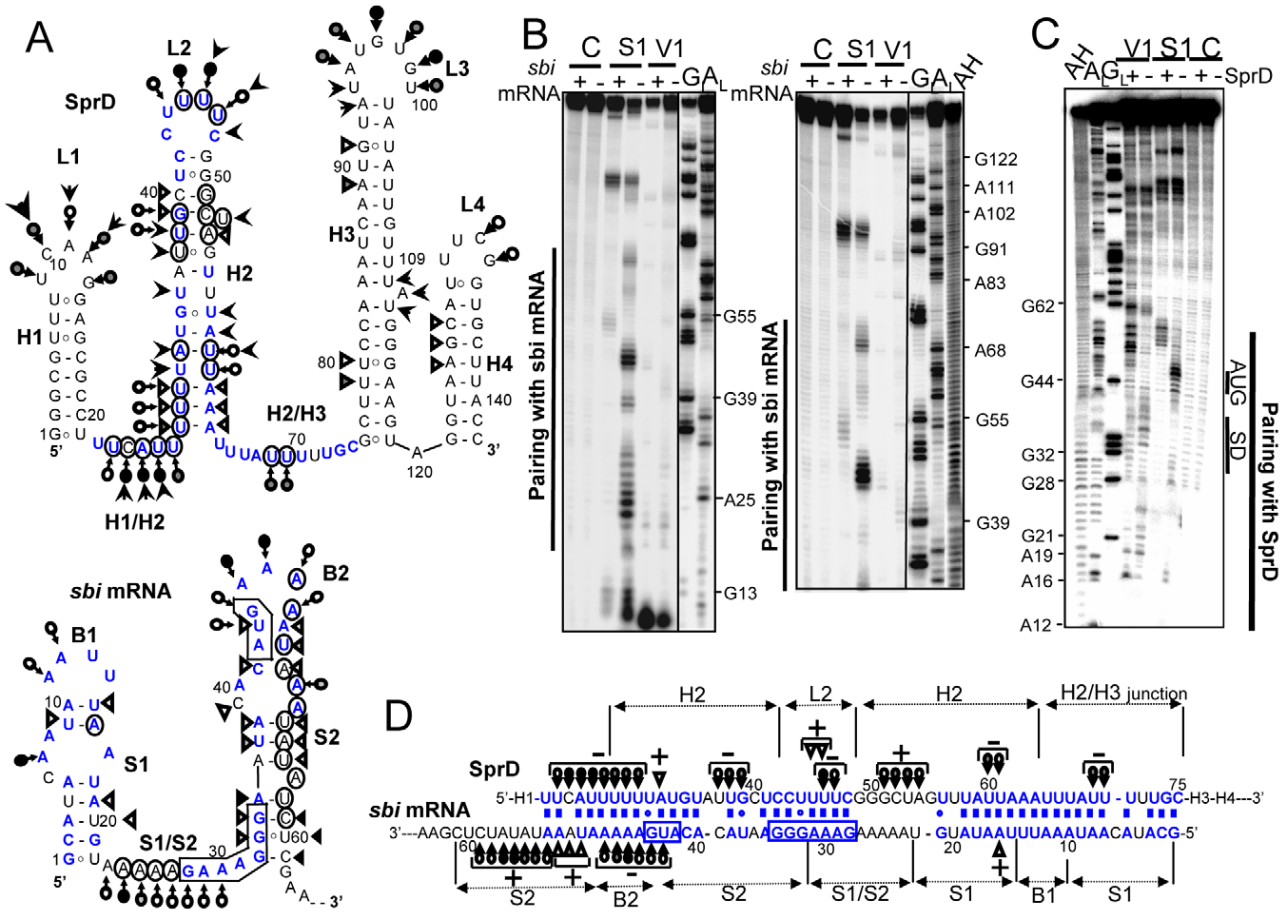
Structural analysis of the ‘SprD-sbi mRNA’ duplex indicates that SprD binds to the *sbi* mRNA ribosome binding site. (**A**) Secondary structures of the SprD RNA and of the *sbi* mRNA 5′-end (nts 1–62) from *S. aureus* N315 based on structural probes in solution that supports each of the proposed structures. Triangles are V1 cuts; arrows capped by a circle are S1 cuts; uncapped arrows are lead cuts. Intensities of cuts and cleavages are proportional to the darkness of the symbols. Structural domains are indicated. The AUG and putative SD sequence are squared on the *sbi* 5′-end mRNA structure. On the secondary structure models of two isolated RNAs, the nucleotides involved in the structural changes induced by the formation of the ‘SprD-*sbi* mRNA’ duplex have been circled. (**B**) Conformational changes of SprD induced by complex formation with the *sbi* mRNA detected by structural probes. Autoradiograms of cleavage products of 5′-labeled SprD by RNases S1 and V1 in the presence (+) or absence (−) of *sbi* mRNA. Lanes C, incubation controls; lanes G_L_, RNase T1 hydrolysis ladder; lanes A_L_, RNase U2 hydrolysis ladder. The RNA sequence is indexed on the right side. (**C**) Conformational changes of the *sbi* mRNA 5′-end induced by complex formation with SprD monitored by structural probes. Indications are as for panel **A**. (**D**) Pairing interactions between SprD and the *sbi* mRNA 5′-end, based on (*i*) computer prediction, (*ii*) native gel retardation assays and mutational analyses, (*iii*) structural mapping of the conformation of SprD in complex with the *sbi* mRNA 5′-end and (*iv*) structural mapping of the conformation of the *sbi* mRNA 5′-end in complex with SprD. Only the structural information concerning the conformation of the duplex is indicated, using similar signs as for panel **A**. The plus (+) and minus (−) signs indicate respectively the appearance or the disappearance of cleavages by the structural probes when the two RNAs are in duplex.

Next, we analyzed in detail complex formation between SprD and the *sbi* mRNA. As a prerequisite to this study, conformations of the free SprD (nt 1–142) and of the 5′-*sbi* mRNA (nt 1–179) were investigated using chemical and enzymatic probes. Both transcripts were end-labeled and their solution structures were probed by RNase V1, which cleaves double-stranded (ds) RNAs or stacked nucleotides, and by nuclease S1 and lead, which both cleave accessible single-stranded (ss) RNAs. The reactivity toward these structural probes were monitored for each nucleotide (**Figure S2** for SprD and **Figure S3** for the 5′-*sbi* mRNA). The data are summarized onto SprD and *sbi* mRNA 5′-end models that they support (**Figure 4A**). Out of the 142 nts of SprD, 96 are involved in intramolecular pairings, implying structural stability. SprD has two folded ends (H1 and H3–H4) flanking a 54 nt-long accessible domain made of an unstable stem (H2) capped by a loop (L2), bordered by two ss (H1/H2 and H2/H3) junctions. For the 5′-end of the *sbi* mRNA, the data support the existence of two folded stem-loops (S1-B1 and S2-B2) flanking a 9 nt-long accessible domain (S1/S2 junction) that contains the predicted SD (Shine-Dalgarno) sequence. The AUG initiation codon is located in loop B2.

The pairing prediction and structural changes induced by complex formation between the two RNAs were examined by subjecting a ‘SprD-*sbi* mRNA’ complex to statistical nuclease S1 and RNases V1 cleavages. Binding of *sbi* mRNA induced structural changes in a restricted region of SprD (from U21 to G76), covering the H1/H2 junction, H2, L2, and the H2/H3 junction (**Figure 4B**). The structural data that supports the interaction within each helix, as drawn in **Figure 4D**, are the following: in the presence of the *sbi* mRNA, S1 cleavages at U23–U30 (H1/H2 junction and H2), U37-G39 and U60–U61 (H2), U46–U47 (loop L2) and A69-U70 (H2/H3 junction) disappeared within the SprD structure, whereas S1 cuts at G51-A54 (H2) appear. Upon duplex formation, V1 cuts appeared at A32 (H2) and at U45–U46 (L2). The binding of SprD led to correlated structural changes in 5′-end of *sbi* mRNA (from G1 to G62, **Figure 4C**), encompassing the predicted SD and AUG initiation codon. In the ‘*sbi* mRNA-SprD’ duplex, S1 cleavages appeared at positions A53-C59 (S2) and disappeared at positions U43-A48 (B2) within the *sbi* mRNA sequence. Also, RNAse V1 cuts at positions A16 and U50-A52 appeared, supporting the complex formation as drawn at **Figure 4D**.

Therefore, these data are consistent with the deletion analysis of the ‘SprD-*sbi* mRNA’ complex and support a bipartite helical interaction between the two RNAs (**Figure 4D**). Structural probing of the RNA duplex indicates that a discontinuous helical domain forms between the two RNAs (22–48_SprD_/28–53_sbi mRNA_ involving the SD and AUG codon and 56–75_SprD_/1–19_sbi mRNA_). This helical domain is interrupted by an accessible ss RNA (49–55_SprD_/22–27_sbi mRNA_). Nucleotides from the *sbi* mRNA flanking the interaction domain (54–59_sbi mRNA_) become heavily cleaved by nuclease S1 due to steric constraints from the neighbouring duplex. These data demonstrate that the interaction between SprD and the *sbi* mRNA involves it's predicted SD sequence and AUG initiation codon.

### The ‘*sbi* mRNA-SprD’ pairing prevents ribosome loading and translation initiation

**Figure 5:**
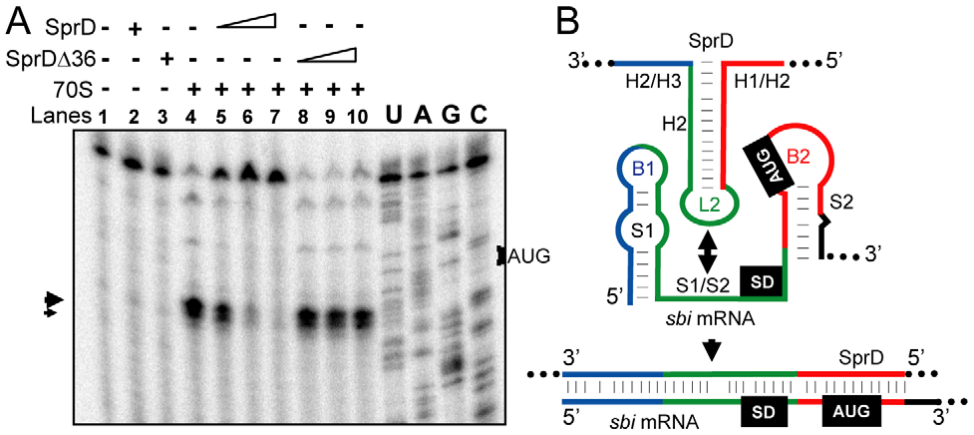
SprD prevents ribosome loading and translation initiation onto the *sbi* mRNA. (**A**) Ribosome toeprints onto the *sbi* mRNA. ‘+/−’ indicates the presence of purified ribosomes with SprD (lanes 2 and 5–7) or with SprDΔ36 (lanes 3 and 8–10). Concentrations of SprD and SprDΔ36 were 0.4 µM (lanes 5 and 8), 2 µM (lanes 6 and 9) and 10 µM (lanes 7 and 10). The experimentally-determined toeprints are indicated with arrows. U, A, G and C refer to the *sbi* mRNA sequencing ladders. (**B**) Schematic view of the antisense regulatory mechanism of SprD with the *sbi* mRNA 5′-end. SprD is proposed to recognize its target mRNA *via* a ‘loop–single strand’ interaction (green) that extends further upstream and downstream.

Since the interaction of SprD with the *sbi* mRNA coincides with the region of mRNAs covered by the ribosomes during translation initiation [22], SprD should prevent ribosome loading on the *sbi* mRNA. To test this, toeprint assays were performed on ternary initiation complexes including purified 70S ribosomes, initiator tRNA^fMet^ and the *sbi* mRNA. Two ribosome toeprints were detected onto the *sbi* mRNA, at 15 and 17 nts downstream from the initiation codon respectively (**Figure 5A**, lane 4), supporting the location of the *sbi* mRNA start codon as drawn on **Figure 4**. SprD reduced ribosome loading onto the *sbi* mRNA in a concentration-dependent manner (**Figure 5A**, lanes 5–7). Increasing amounts of SprDΔ36, that cannot form a complex with the *sbi* mRNA (**Figure 5B**), did not prevent ribosome loading onto the *sbi* mRNA (**Figure 5A**, lanes 8–10). It is concluded that SprD inhibits *sbi* mRNA translation by preventing ribosome binding by antisense pairings with the *sbi* mRNA 5′-end. These results are in agreement with data obtained *in vivo*, showing that SprD inhibits Sbi expression at translational level.

### SprD enhances the virulence of *S. aureus*

**Figure 6:**
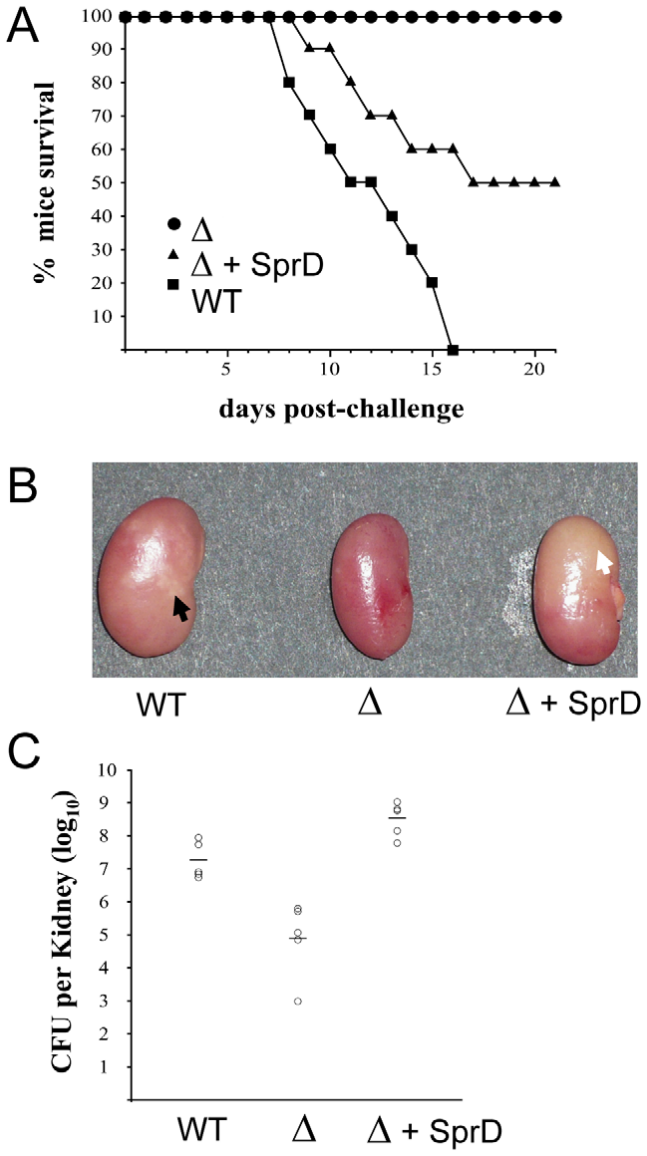
The SprD RNA enhances the virulence of a *S. aureus* clinical isolate on infected mice. (**A**) Survival of mice infected with *S. aureus* wild-type strain N315 (black square), its isogenic Δ*sprD* mutant (black circle) and Δ*sprD* mutant complemented with pCN38ΩsprD (black triangle). Groups of 10 eight-week old Swiss mice were inoculated i.v. with 10^9^ bacteria and monitored daily for 3 weeks. (**B**) Macroscopic aspect of kidneys after i.v. infection with *S. aureus* wild-type strain N315 (WT), isogenic Δ*sprD* mutant (Δ) and Δ*sprD* mutant complemented with pCN38ΩsprD (Δ+SprD). Increased size, discoloration and multiple abscesses (black arrow) caused by the wild-type strain was not observed with the Δ*sprD* mutant, while the Δ*sprD* complemented strain yielded diffuse discoloration instead of focal abscesses (white arrow). Eight-week old Swiss mice were inoculated with ca. 1.5×10^8^ bacteria and sacrificed after six days. (**C**) Recovery of *S. aureus* strains from the kidneys of infected mice six days after bacterial challenge. Groups of 5 mice were inoculated i.v. with ca. 1.5×10^8^ CFU of wild-type strain N315, Δ*sprD* mutant and Δ*sprD* mutant complemented with pCN38ΩsprD, respectively. Each individual animal is indicated by a circle symbol with mean bacterial titres represented as a line.

Since one SprD target is the Sbi immune-evasion molecule that was proposed to be involved in *S. aureus* pathogenicity [4], [5], this RNA may play (a) role(s) during staphylococcal infections. This suggestion is in agreement with its co-location with virulence factors [12]. Therefore, we tested the importance of the SprD RNA during staphylococcal infections on an animal infection model. Using a murine i.v. sepsis model with an inoculum of 10^9^ *S. aureus* per mouse, we showed that the virulence of the Δ*sprD* mutant is abolished (100% survival at day 21 of infection), whereas all animals infected with the parental wild-type strain die (**Figure 6A**). The virulence of the *trans*-complemented Δ*sprD*+SprD strain is partially restored as compared to the wild type (50% survival at day 21, *P*<0.02). In a different i.v. infection experiment with a 5×10^8^ CFU inoculum per mouse in which animals were sacrificed at day 6, the kidneys of mice inoculated with the Δ*sprD* mutant are small and homogenous red-brown, whereas those of mice inoculated with the wild-type strain are substantially swollen and displayed mottled discoloration suggesting numerous abscesses (**Figure 6B**). Kidneys of mice infected with the Δ*sprD*+SprD strain are slightly less swollen than the latter, but display homogenous discoloration with no distinct abscesses (**Figure 6B**). Results of the macroscopic observation are confirmed in the same experiment by viable bacteria counts, as the mean kidney titres (± SD) were 7.2±0.3, 4.9±1.0, 8.5±0.6 log_10_ CFU per pair of kidneys for the wild-type, Δ*sprD*, and Δ*sprD*+SprD strains, respectively (**Figure 6C**). After 6 days of infection, the *in vivo* persistence of plasmid pCN38Ω*sprD* in the Δ*sprD*+SprD strain was verified in 160 randomly selected colonies obtained from kidney homogenates. All of them have retained resistance to chloramphenicol, a specific marker of pCN38. The virulence defect of a SprD-deletion strain, compared to an isogenic wild-type strain, was also observed in the *agr* positive RN1 strain (data not shown). Altogether, these results demonstrate the importance of SprD during bacterial infections triggered by *S. aureus* clinical isolates. Using the same murine i.v. sepsis model, we also tested the implications of Sbi in *S. aureus* virulence. For this purpose, a *sbi* deletion strain (Δ*sbi*) and a strain overexpressing *sbi* under its endogenous promoter from the pCN35Ω*sbi* plasmid (*sbi^+^*) were constructed (**Figure panels S6A and S6B**). We showed that the virulence of the two Δ*sbi* and *sbi^+^* mutants is similar to that of the isogenic wild-type strains (**Figure S6C**). These results indicate that only varying the expression levels of the Sbi protein is insufficient to account for the SprD virulence phenotype in our animal infection model and imply that SprD has additional target(s) involved in staphylococcal virulence. Taken together, our findings indicate that SprD plays a major role in the virulence of *S. aureus*.

## Discussion

In this report, we show that a small regulatory RNA expressed by *S. aureus* clinical strains plays an essential role in bacterial virulence during the infection of mice in a model of septicaemia. After RNAIII, SprD is the second regulatory RNA that plays a major role in *S. aureus* virulence. RNAs are emerging as regulators that enable bacterial pathogens to express virulence genes when required during infection, illustrating their essential roles in pathogenesis [23]. Numerous sRNAs are implicated in the infections caused by Gram-positive and negative bacteria [23]. Some sRNAs are expressed from pathogenicity islands [12], and such horizontally acquired post-transcriptional regulators can regulate the expression of genes encoded by the core genome [24 and this report]. Some sRNAs regulate the expression of virulence factors [10] or are expressed when bacteria multiplies within mammalian cells [25]. Their implication in bacterial pathogenesis, however, was not demonstrated in animal models of infection. Recent studies have shown that several sRNAs expressed from various bacteria including *V. cholerae*, *L. monocytogenes* and *S. typhimurium* modulate or are involved in virulence on mice infection models [26]–[28].

In *S. aureus*, RNAIII is the paradigm for RNA-controlled expression of virulence genes, being the effector of the *agr* system. RNAIII was the first RNA shown to be involved in bacterial pathogenesis more than fifteen years ago [9] and is the only example in *S. aureus* until now. Compared to the 142 nt-long SprD, the RNAIII (514 nt-long) is almost four times bigger, encodes a small protein, has a complex structure made of 14 stem-loops [29] and regulates the expression of several virulence genes [10]. The importance of *agr* for virulence in animal models has been reported [30]–[31], but the exact contribution of RNAIII awaits the experimental testing of an RNAIII deletion strain.

This report reveals that a small regulatory RNA expressed by *S. aureus*, SprD, enhances the virulence of the *agr* negative N315 clinical strain (**Figure 6**) and of the *agr* positive RN1 strain (data not shown). All the mice infected with the *S. aureus* strain that does not express SprD survive three weeks after the inoculation, whereas all mice challenged with the wild type strain expressing SprD die within 16 days following inoculation. The virulence of the *trans*-complemented strain is half restored, with the mice kidneys containing viable bacteria as for the wild type strain. The partial restoration of the virulence of the complemented strain could be due to partial plasmid loss after day 6 or, on the other hand, to a negative impact on bacterial virulence of the higher expression of SprD from the plasmid, compared to the wild type strain. The macroscopic aspect of kidneys from mice infected with bacteria expressing, or not, SprD as well as the lower amounts of bacteria detected in the infected kidneys when SprD is not expressed, indicate that this RNA plays a major role in the virulence of *S. aureus* (Figure 6). The effect of SprD on virulence might be linked to the lower amount of bacteria detected in the infected kidneys in the absence of the RNA.

We tested the ability of SprD to modify gene expression in *S. aureus* cells and identified the immune evasion Sbi protein as one molecular target of the RNA. The Sbi protein is among the most abundant secreted proteins [32] produced by many *S. aureus* clinical isolates [4], [33]. We have unravelled the mechanism by which SprD regulates Sbi expression. The action of SprD on the *sbi* mRNA proceeds by antisense pairings, blocking translation initiation. The pairing interaction between SprD and the *sbi* mRNA and its functional outcome is presented as a model in **Figure 5B**. A central domain of SprD pairs with the *sbi* mRNA 5′-end that includes its SD sequence and AUG initiation codon, blocking translation initiation. For SprD, all the structural changes induced by the formation of the duplex are located in stem-loop H2 and single-stranded flanking domains H1/H2 and H2/H3. The pairings between SprD and the *sbi* mRNA could be divided into three interacting domains that include the very 5′-end of the *sbi* mRNA, its SD sequence and its AUG initiation codon. The interacting domains that are single-strand in each of the two RNA structures probably pair first (the H2/H3 junction_SprD_ with B1_sbi_, L2_SprD_ with the purine-rich S1/S2 junction_sbi_ and the H1/H2 junction_SprD_ with B2_sbi_), followed by spreading through their respective secondary structures. *In vitro* and *in vivo*, experimental evidences demonstrate that the regulation of Sbi expression by SprD takes place without the need of the Hfq protein, illustrating the facultative requirement of the Hfq protein for sRNA–mRNA duplex formation among bacteria. The ‘SprD-*sbi* mRNA’ interaction involves 41 base-pairs and, as suggested [34], extended pairings probably overcome the requirement for the Hfq RNA chaperone.

This strategy of gene expression inhibition is frequently used by bacterial regulatory RNAs [reviewed in 21], including the downregulation of another IgG binding protein, SpA, by the RNAIII [35]. Translation inhibition by regulatory RNAs in bacteria is usually sufficient for gene silencing and can occur in the absence of mRNA destabilization [36]. If target mRNA degradation is triggered, as with the double-strand specific RNAse III in some RNA-mediated gene regulations in *S. aureus* [35], the process of gene silencing becomes irreversible. SprD does not affect the *sbi* mRNA levels, indicating that this gene regulation could be reversible.

In this report, we demonstrate that Sbi is directly regulated by SprD *in vivo* and *in vitro* and we also show that this SprD-mediated regulation is *agr* independent. Indeed, it was previously reported that the inactivation of the *agr* global virulence regulator increases the abundance of Sbi *in vivo* [32], indicating that *agr*, as SprD, is a negative regulator of Sbi expression. We show that SprD regulates the expression of the Sbi protein in both *agr* positive (SH1000, RN4220 and RN1) and *agr* negative (N315) strains, demonstrating that the SprD-mediated regulation of Sbi occurs independently of *agr*. In addition, in various clinical strains, Sbi expression is induced by human IgGs [37] although the mechanism of such a positive regulation is currently unknown, but is independent of the RNAIII (data not shown). IgGs increase the levels of the Sbi protein in the presence and absence of SprD (**Figure S4**), indicating that the two regulations are independent. Hence, the Sbi expression is monitored by at least three regulatory pathways, suggesting that the amount of Sbi has to be precisely controlled in *S. aureus* cells. Such a sophisticated regulation network implies that this protein should be an important factor for staphylococcal physiology.

The Sbi protein interferes with innate immune recognition by binding multiple host proteins including the complement factors H and C3 as well as IgG (the Sbi protein traps human IgGs [4]) and β2-glycoprotein I [5], [38], [39], suggesting that Sbi has a role during staphylococcal infections. Analysis of the virulence of *sbi* deletion and overexpression strains suggests that Sbi does not appear to be a major virulence factor for staphylococcal infection in a model of septicaemia. Similarly to SpA, the first discovered staphylococcal immunoglobulin-binding protein which has properties comparable to those of Sbi [40], its contribution to bacterial virulence was difficult to prove *in vivo*, demonstrating the variability of the results obtained depending on the animal model considered [41]–[43]. As for SpA, the effect of Sbi on virulence is probably hard to be identified, only visible in a few infection models. Since the Sbi protein is predicted to be implicated at early stages of the infection, its contribution is difficult to assess in our infection model. Moreover, the Sbi and SpA proteins could have overlapping functions in host immune evasion, deregulation of expression of either *sbi* or *spa* may be insufficient to induce virulence defects on animal models. The *sbi* deletion or the Sbi overproduction have no detectable virulence phenotypes in our infection model, indicating that the virulence defect of the *sprD* deletion mutant is not caused only by the deregulation of the Sbi expression levels. Thus, SprD is predicted to have other target(s) and/or more general functions implicated in staphylococcal virulence. We do not exclude, however, the implication of Sbi in *S. aureus* virulence.

The expression profile of SprD during growth shows elevated expression levels at stationary phase (**Figure 1**) when the *sbi* mRNA levels are sparse (**Figure 2E**), implying that SprD functions are not restricted to the regulation of Sbi expression, also suggesting that SprD has additional target(s) that could be involved at various times during the infection. Indeed, RNAs often regulate the expression of more than a single target, as for several *E. coli* RNAs [reviewed in 44] and for the *S. aureus* RNAIII [11]. Also, it would not be so surprising that regulatory RNA(s) other than SprD act synergistically to regulate the expression of the sbi mRNA during cell growth, and a reasonable candidate could be the RNAIII. As for SprD that regulates the expression of Sbi and of other putative target(s), the RNAIII represses, by antisense pairings, the expression of the Sbi-like SpA protein and also controls the expression of additional genes either directly or by limiting the expression of the Rot transcriptional regulator [11]. Preliminary data obtained in our laboratory indicate that SprD has at least one mRNA target in *S. aureus* cells. The identification of SprD additional target(s) and learning how they are regulated by SprD will be required to understand implication of this sRNA in *S. aureus* virulence. Identification of Sbi as the first target of SprD is an important step in elucidating the complete gene network regulated by this small RNA which has such a major role in virulence.

Our work, in combination with what is known about RNAIII, suggests a major role for regulatory RNAs in *S. aureus* pathogenicity. This study also illustrates how sophisticated the regulations of virulence factors productions are during *S. aureus* infections. It reinforces the roles of RNAs in regulating numerous biological processes in this bacterium. Further studies will be necessary to identify the complete gene network regulated by SprD, its additional target(s), why SprD has such an important role in staphylococcal virulence and the underlying mechanisms of regulations.

## Materials and Methods

### Strains and plasmids

Strains and plasmids are listed in **Table S2**. *S. aureus* trains were cultured at 37°C in brain heart infusion broth (BHI, Oxoid). When necessary, chloramphenicol and erythromycin were used at a 10 µg/ml concentration. In pCN38Ω*sprD* and pCN35Ω*sprD sprD* is expressed from its own promoter. The *sprD* sequence with 40 nts upstream and 35 nts downstream was amplified from N315 genomic DNA as a 217-bp fragment, with flanking *Pst*I and *Eco*RI sites. The PCR product was cloned in pCN38 [45] and pCN35 [45]. For producing the pCN38*ΩsprDΔ36*, mutagenized oligonucleotides ‘T7sprD_delfor’ and ‘T7sprD_delre’ were used (**Table S3**). In pCN35Ω*sbi*, *sbi* is expressed from its endogenous promoter. The *sbi* sequence was PCR amplified from N315 genomic DNA as a 1700-bp fragment with flanking *Pst*I and *Eco*RI restriction sites.

### Construction of the deletion strains

To inactivate the *sprD* gene, DNA fragments of 1000 bp upstream and 800 bp downstream of *sprD* were amplified by PCR from genomic DNA and cloned together with the *ermB* from pCN51 [45] into *Xba*I-*Eco*RI sites of temperature-sensitive plasmid pBT2 [46]. Primers used for cloning are indicated in **Table S3**. The resulting plasmid pBT2Δ*sprD* was transformed into *S. aureus* strain RN4220 and then into *S. aureus* N315 to achieve integration of the *ermB* gene into the genome by homologous recombination. Mutants were enriched by cultivation at 42°C. Cells from the stationary-phase culture were plated on TSA plates and incubated at 37°C. Colonies were imprinted on plates supplemented with 10 µg/mL chloramphenicol. Chloramphenicol-sensitive colonies were tested by PCR for replacement of *sprD* for the erythromycin cassette. The deletion of *sprD* was confirmed by Northern blot (**Figure 2A**). Inactivations of the *sbi* and *RNAIII* genes were performed by the same method except that no resistance marker was inserted between their 5′ and 3′ DNA sequences. The primers used for constructing pBT2Δ*sbi* and pBT2Δ*RNAII* are shown in **Table S3.**

### Animal infection model

Virulence levels of the SprD+ strain N315, its isogenic mutant Δ*sprD* and complemented strain Δ*sprD* pCN38Ω*sprD* were compared using a murine intravenous sepsis model. Groups of 10 female Swiss mice, 6- to 8-weeks old (Charles River Laboratories, L'Arbresle, France) were inoculated i.v. with 300 µL of bacterial suspensions containing 10^9^ *S. aureus* cells in 0.9% NaCl. The survival of the mice was monitored for 21 days, and the statistical significance of differences between groups was evaluated using the Mann-Whitney U test. A *P* value of <0.05 was considered significant. With the same three strains, 3 groups of 5 female Swiss mice, 6- to 8-weeks old (Charles River Laboratories) were then infected i.v. with 5×10^9^ bacteria. Six days after inoculation, the mice were euthanized with CO_2_ and their kidneys excised. After photographs were taken, the organs were homogenized, diluted in 0.9% NaCl and plated on 5% blood agar for determination of bacterial titres, expressed as log_10_ CFU per pair of kidneys. Morphology observation included swelling, discoloration and presence of macroscopic abscesses. The stability of plasmid pCN38Ω*sprD* (encoding chloramphenicol resistance) in the complemented Δ*sprD* mutant was assessed by plating randomly selected colonies grown from kidney homogenates on nutrient agar with containing 20 µg/mL chloramphenicol.

### Protein isolation, mass spectrometry and immunoblots

For the preparation of protein extracts, bacteria are grown until the exponential or stationary phases and the cells are pelleted for 10 min at 4°C (8.000g). For purifying the extracellular proteins, the supernatants are collected, filtered (0.45 µm sterilized filter) and precipitated with 10% trichloroacetic acid. The precipitates are washed with ice-cold acetone and loaded onto SDS-PAGE according to [47]. For the total protein extractions, pellets of 2-ml cultures are washed with TE (50 mM EDTA, 50 mM Tris pH 7.5), and suspended in 0.2 ml of the same buffer containing 0.1 mg/ml lysostaphin. Following incubation at 37°C for 10 min, samples are boiled for 5 min, analyzed by SDS-PAGE and stained by Coomassie blue R-250. The proteins of interest are extracted from gel, trypsin digested and the peptides identified by MALDI MS/MS and RP-HPLC/NanoLC/ESI-MS-MS. For the immunoblots, proteins are transferred to PVDF membrane (Immobilon-P, Millipore). Signals are visualized using a STORM 840 Phosphor-Imager (Molecular Dynamics) and quantified using Image-QuantNT 5.2.

### RNA isolation, Northern blots, 5′- RACE, transcription and RNA labeling

Total RNAs are prepared as described [48]. For SprD and other sRNAs, Northerns are performed with 5 µg of total RNAs, as described [12]. For *sbi* mRNA, Northerns are performed as described [49]. RACE assays are carried out according to 49 with the primers from **Table S3**. Wild-type and mutant RNAs for probing, gel-shift assays or toeprints are transcribed from PCR fragments generated from genomic DNA with the primers from **Table S3**. For producing the template-encoding SprDΔ36, mutagenized oligonucleotides (**Table S3**) were used. The RNAs were produced by *in vitro* transcription using MEGAscript (Ambion). Adding [α^32^-P]UTP within the transcription mix produces radioactive transcripts. 5′-RNA labeling is performed as described [49]. The RNAs are purified by 8% PAGE, eluted, ethanol precipitated and stored at −80°C.

### Gel-shift assays and RNA probing

Gel retardation assays are performed as described [49], 0.4 pmol of labeled wt or *sbi*Δ61 mRNAs are incubated with various concentrations (from 1.6 to 20 pmols) of unlabeled wt SprD or SprDΔ36. For structural analysis duplexes between *sbi* mRNA and SprD are prepared by incubating 0.4 pmol of labeled RNA and 1.6 pmol of unlabeled RNA in a buffer containing 10mM Tris-HCl (pH 7,5), 60 mM NaCl, 10mM EDTA and 5 mM DTT for 15 min at 25°C. Structural assays are performed as described [49]. Digestions are at 25°C for 15 min with 2.5 µg of *yeast* tRNAs with 0.2 or 1 unit of S1 and 10^−4^ or 5.10^−5^ units of V1. Lead(II) cleavages are performed with 0.2 or 0.4 mM PbAc in 25 mM Hepes (pH 7.5), 7 mM Mg acetate and 35 mM K acetate for 10 min at 25°C. The reactions are precipitated, the pellets dissolved in loading buffer (Ambion). The samples are denatured for 5 min at 65°C prior to separation on 8% polyacrylamide/8M urea gels. Gels are dried and visualized (STORM 840 Phosphor-Imager).

### Toeprints

The toeprints are as described [50] with modifications. Annealing mixtures contain 0.2 pmol of *sbi* mRNA and 1 pmol of labeled ‘SBIrevTR’ primer in a buffer containing 10 mM Tris-acetate (pH 7.5), 60 mM NH_4_Cl, and 1 mM DTT. For the assays in the presence of SprD, various concentrations of wt or SprDΔ36 are added prior to the purified *E. coli* 70S ribosomes. The ribosomes are reactivated for 15 min at 37°C and diluted in the reaction buffer in the presence of 1 mM MgCl_2_. 4 pmols of 70S are added in each assay, incubated for 5 min and MgCl_2_ is adjusted to 10 mM. After 5 min, 10 pmols of uncharged tRNA^fMet^ are added and incubated for 15 min. cDNA is synthesized with 2 UI of AMV RT (Biolabs) for 15 min. Reactions are ended by 10 µl of loading buffer II (Ambion). The cDNAs are loaded and separated onto 8% PAGE. The toeprints are located on the *sbi* mRNA sequence by sequencing the DNA.

### Ethics statement

All animal experiments were performed in accordance to European guidelines and recommendation of the French Agricultural Office for the care of animals subjects. Experiments were carried out in the accredited research animal facility of Institut Pasteur de Lille (accreditation number, A59107). All animal protocols were approved by the locally appointed investigational review board (Institut Pasteur de Lille, accreditation number, A59107).

### List of accession numbers of genes and proteins mentioned in the text

*S. aureus* Immunoglobulin G binding protein A : GenBank ID: BAB41326.1

*S. aureus* Sbi protein: Genbank ID: AF027155

*S. aureus* RNAIII (nt 1260 to 1571): GenBank accession number: X52543

*S. aureus* Hfq: PDB code 1Kq1A

## Supporting Information

Figure S1 Sequence alignments of SprD from several *S. aureus* strains. The bolded nucleotides are the 5′-ends derived from N315 RACE mapping and the underlined nucleotides are the sequence variations. The stars are the sequence identities. SprD has a 9- base pair helix (H4) ending by a U_6_ stretch, acting as a transcription terminator.
(0.06 MB DOC)

Figure S2 Monitoring of SprD conformation by structural probes. SprD conformation was probed by RNase V1, that cleaves double-strands (ds) or stacked nucleotides and by nuclease S1 and lead that both cleave accessible single-strands (ss). Autoradiograms of cleavage products of 5′-labeled SprD by lead, nucleases S1 (0.2 or 1 unit) and V1 (10^−4^ or 5.10^−5^ unit) from long (left) and short (right) runs. Lanes C, incubation controls; Lanes G_L_, RNAse T1 hydrolysis ladders; lanes A_L_, RNAse U2 hydrolysis ladder; lanes AH, alkaline hydrolysis ladders. The sequence is indexed at the right side. The reactivity toward these probes was monitored for each nucleotide. Ds-specific cuts at U28–U30, G39–C40, A54, A62–A64, U79–U80, A89, G91 and A125–A127 and the absence of nuclease S1 and lead cleavages at G1-U8, G14-U21, C40–C42, G49-C52, G76-U92, U101–U109, G113-U119, G121-U129 and G134-C142 indicate that four RNA helices (H1–H4) form (Fig. 1A). Helix H2 can be extended from 4 to 13 base-pairs, but its lower portion is cut by both ss- (U28-G39, G55-A64) and ds- (U28–U30, A62–A64) probes, implying instability and breathing. S1 cuts at U9-G13, U44-C48, A95-U100, C132-G133 and lead cuts at A11–A12, U46-C48 and U94 support loops L1–L4. Based on S1 and lead cleavages and no V1 cuts, U22–U27 and U65-C75 fold as an ss RNA. Lead cuts at U110–U112 support an internal bulge within H3.
(2.84 MB DOC)

Figure S3 Monitoring the conformation of the *sbi* mRNA 5′-end (179 nts) by structural probes. Autoradiograms of cleavage products of 5′-labeled *sbi* mRNA by RNase V1, nuclease S1 and lead. For the details, please refer to Figure S3 legend.
(6.50 MB DOC)

Figure S4 Human IgGs from serum increase Sbi protein levels in the presence (+) and absence (−) of SprD. Immunoblot analysis with anti-Sbi antibodies of total intracellular proteins in *S. aureus* SH1000 strain in the presence (+) or absence (−) of 10% human serum.
(0.05 MB DOC)

Figure S5 Coomassie staining of the samples presented on Fig. 2, panels B and C (A) and on Fig. 2D (B) indicates that identical amounts of proteins were loaded for strains ‘wt’, ‘Δ’ and Δ+sprD'.
(0.52 MB DOC)

Figure S6 Deleting or overproducing the Sbi protein have no detectable effect on the virulence of the N315 *S. aureus* clinical isolate on infected mice. Monitoring the expression of the Sbi protein in strains N315 Δsbi (A) and in the sbi overproducing strain pCN35-sbi (B), compared to a strain carrying the empty plasmid vector (pCN35) and to the wild-type strain (wt) by immunoblots with anti-Sbi antibodies. (C) Survival of mice infected with *S. aureus* wild-type strain N315 (square), its isogenic Δsbi mutant (circle) and wild-type strain transformed with pCN35Ωsbi (triange). Groups of 5 seven-week old Swiss mice were inoculated i.v. with 2.109 bacteria and monitored daily for 2 weeks.
(2.48 MB DOC)

Table S1 MS identification of the Sbi protein by detecting 25 Sbi peptides.
(0.06 MB DOC)

Table S2 Strains and plasmids used in this study.
(0.05 MB DOC)

Table S3 DNA primers used in this study.
(0.08 MB DOC)
