# Supplementary figures and images for: A Staphylococcus aureus Small RNA Is Required for Bacterial Virulence and Regulates the Expression of an Immune-Evasion Molecule
Source: PLoS Pathog. 2010 Jun 3;6(6):e1000927. doi: 10.1371/journal.ppat.1000927 (PMC2880579; doi:10.1371/journal.ppat.1000927)

**
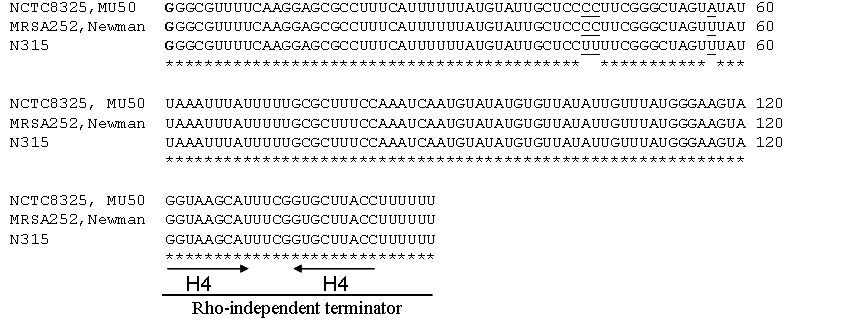
**

Supplement: Figure S1 — Sequence alignments of SprD from several S. aureus strains. The bolded nucleotides are the 5′-ends derived from N315 RACE mapping and the underlined nucleotides are the sequence variations. The stars are the sequence identities. SprD has a 9- base pair helix (H4) ending by a U6 stretch, acting as a transcription terminator. (0.06 MB DOC) [file ppat.1000927.s001.doc]

**
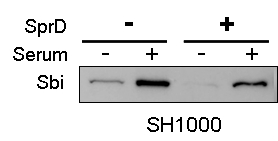
**

Supplement: Figure S4 — Human IgGs from serum increase Sbi protein levels in the presence (+) and absence (−) of SprD. Immunoblot analysis with anti-Sbi antibodies of total intracellular proteins in S. aureus SH1000 strain in the presence (+) or absence (−) of 10% human serum. (0.05 MB DOC) [file ppat.1000927.s004.doc]

A B


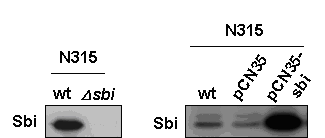


C

Supplement: Figure S6 — Deleting or overproducing the Sbi protein have no detectable effect on the virulence of the N315 S. aureus clinical isolate on infected mice. Monitoring the expression of the Sbi protein in strains N315 Δsbi (A) and in the sbi overproducing strain pCN35-sbi (B), compared to a strain carrying the empty plasmid vector (pCN35) and to the wild-type strain (wt) by immunoblots with anti-Sbi antibodies. (C) Survival of mice infected with S. aureus wild-type strain N315 (square), its isogenic Δsbi mutant (circle) and wild-type strain transformed with pCN35Ωsbi (triange). Groups of 5 seven-week old Swiss mice were inoculated i.v. with 2.109 bacteria and monitored daily for 2 weeks. (2.48 MB DOC) [file ppat.1000927.s006.doc]
