# Supplementary material for: A Staphylococcus aureus Small RNA Is Required for Bacterial Virulence and Regulates the Expression of an Immune-Evasion Molecule
Source: PLoS Pathog. 2010 Jun 3;6(6):e1000927. doi: 10.1371/journal.ppat.1000927 (PMC2880579; doi:10.1371/journal.ppat.1000927)
Supplement: Table S1 — MS identification of the Sbi protein by detecting 25 Sbi peptides. (0.06 MB DOC) [file ppat.1000927.s007.doc]

**Table S1.** Mass spectrometry identification of the Sbi protein by detecting 25 Sbi peptides

| **Observed** | **Mr (expected)** | **Mr (calculated)** | **Peptides** |
| --- | --- | --- | --- |
| 452.2365 | 902.4584 | 902.4385 | K.YLTDTYK.S |
| 493.2919 | 984.5692 | 984.5604 | K.QLDALVAQK.D |
| 539.7876 | 1077.5606 | 1077.5454 | K.AIKDFQDNK.A |
| 542.8126 | 1083.6107 | 1083.5964 | K.LLGYYQSLK.D |
| 559.8298 | 1117.6450 | 1117.6284 | K.AFYQVLHLK.G |
| 561.7985 | 1121.5825 | 1121.5716 | K.VDDKNGYLAK.S |
| 562.8242 | 1123.6338 | 1123.6237 | K.VEVPQIQSPK.V |
| 565.8000 | 1129.5855 | 1129.5801 | R.EVNKAPMDVK.E |
| 566.2913 | 1130.5680 | 1130.5641 | R.EVNKAPMDVK.E + Deamidation (NQ) |
| 569.3008 | 1136.5871 | 1136.5713 | R.AQEVFSESLK.D |
| 589.2702 | 1176.5259 | 1176.5127 | K.YYYNTYYK.Y |
| 609.3096 | 1216.6047 | 1216.6047 | K.LNEKDSIENR.R |
| 613.3108 | 1224.6071 | 1224.5986 | R.NYVTESINTGK.V |
| 639.8308 | 1277.6470 | 1277.6615 | K.SAAYEANSKLPK.D |
| 653.3601 | 1304.7057 | 1304.6976 | K.SYIQPLKVDDK.N |
| 697.8796 | 1393.7447 | 1393.7353 | R.VAQQNAFYNVLK.N |
| 734.3853 | 1466.7560 | 1466.7252 | R.AQEVFSESLKDSK.N |
| 746.3830 | 1490.7515 | 1490.7365 | R.SQQVWVESVQSSK.A |
| 773.4359 | 1544.8572 | 1544.8409 | K.GAIDQTVLTVLGSGSK.S |
| 775.9313 | 1549.8481 | 1549.8364 | R.RVAQQNAFYNVLK.N |
| 793.4562 | 1584.8978 | 1584.8551 | K.VLYTFYQNPTLVK.T |
| 846.4664 | 1690.9183 | 1690.9253 | K.VEAPQIQSPQIEKPK.A |
| 852.9039 | 1703.7933 | 1703.7863 | K.HQTTQNNYVTDQQK.A |
| 919.0168 | 1836.0191 | 1835.9992 | K.YKGAIDQTVLTVLGSGSK.S |
| 1018.5161 | 2035.0177 | 2034.9857 | K.NDNLTEQEKNNYIAQIK.E |
