# Supplementary material for: A Staphylococcus aureus Small RNA Is Required for Bacterial Virulence and Regulates the Expression of an Immune-Evasion Molecule
Source: PLoS Pathog. 2010 Jun 3;6(6):e1000927. doi: 10.1371/journal.ppat.1000927 (PMC2880579; doi:10.1371/journal.ppat.1000927)
Supplement: Table S2 — Strains and plasmids used in this study. (0.05 MB DOC) [file ppat.1000927.s008.doc]

| **Strains** | **Relevant characteristics** | **References** |
| --- | --- | --- |
| ***E. coli strain*** |  |  |
| DH5α | F^-^ φ80 Δ*lacZ ΔM15 Δ*(*lacZA-argF*)*U169 deoR recA1 endA1 hsdR17*  (r_K_^-^ m_K_^-^) *phoA supE44* λ^-^ *thi-1 gyrA96 relA1* | 1 |
| ***S.aureus strains*** |  |  |
| RN1HG | rsbU restored strain 8325, lysogenic for phages *φ*11, *φ*12, and *φ*13 | 2 |
| RN4220 | Restriction-defective derivative of 8325-4 | 3 |
| RN4220Δ*hfq* | RN4220 deleted for *hfq* | 4 |
| SH1000 | Functional *rsbU* derivative of 8325-4 *rsbU* | 5 |
| N315 | Meticillin-resistant *S aureus* (MRSA) strain isolated in 1982 from the pharyngeal smear of a Japanese patient | 6 |
| MRSA252 | Meticillin-resistant wild type clinical isolate | 7 |
| N315 *∆sprD* | N315 deleted for *sprD* | This study |
| N315 *∆sbi* | N315 deleted for *sbi* | This study |
| ***Plasmids*** |  |  |
| pCN38 | Low-copy-number shuttle vector with Amp^r^ in *E. coli* and Em^r^ in *S. aureus* | 8 |
| pCN38Ω*sprD* | pCN38 with *sprD* under the control of its endogenous promoter | This study |
| pCN38Ω*sprDΔ*36 | pCN38 with *sprDΔ*36 under the control of its endogenous promoter | This study |
| pCN35 | High-copy-number shuttle vector with Amp^r^ in *E. coli* and Em^r^ in *S. aureus* | 8 |
| pCN35Ω*sprD* | pCN35 with *sprD* under the control of its endogenous promoter | This study |
| pCN35Ω*sbi* | pCN35 with *sbi* under the control of its endogenous promoter | This study |
| pBT2 | Low-copy-number shuttle vector with Amp^r^ in *E. coli* and temperature-sensitive replication with Cm^r^ in *S. aureus* | 9 |
| pBT2Δ*sprD* | pBT2 vector with nonpolar erythromycin resistance cassette ligated in-between the 1000-bp upstream region of *sprD* and the 800-bp downstream region of *sprD* | This study |
| pBT2Δ*sbi* | pBT2 vector containing the 940-bp upstream genomic sequence of *sbi* and its 1060-bp downstream sequence, excluding the *sbi* gene sequence | This study |
| pBT2Δ*RNAIII* | pBT2 vector containing the 850-bp upstream genomic sequence of *RNAIII* and its 1000-bp downstream sequence, excluding the *RNAIII* gene sequence | This study |

**Table S2.** Strains and plasmids used in this study

**REFERENCES**

1. Sambrook J, Firtsch EF and Maniatis T (1989) Molecular Cloning: A Laboratory Manual. Cold Spring Harbour Laboratory Press, New York.

2. Pohl K et al. (2009) CodY in Staphylococcus aureus: a regulatory link between metabolism and virulence gene expression. J. Bacteriol. 191: 2953–2963.

3. Kreiswirth BN et al. (1983) The toxic shock syndrome exotoxin structural gene is not detectably transmitted by a prophage. Nature 305: 709-712.

4. Bohn C, Rigoulay C and Bouloc P (2007) [No detectable effect of RNA-binding protein Hfq absence in Staphylococcus aureus.](http://www.ncbi.nlm.nih.gov/pubmed/17291347?ordinalpos=1&itool=EntrezSystem2.PEntrez.Pubmed.Pubmed_ResultsPanel.Pubmed_DefaultReportPanel.Pubmed_RVDocSum) BMC Microbiol. 7.

5. Horsburgh MJ et al. (2002) SigmaB modulates virulence determinant expression and stress resistance: characterization of a functional rsbU strain derived from Staphylococcus aureus 8325-4. J. Bacteriol. 184: 5457-5467.

6. Kuwahara-Arai K, Kondo N, Hori S, Tateda-Suzuki E, Hiramatsu K (1996) Suppression of methicillin resistance in a mecA-containing pre-methicillin-resistant Staphylococcus aureus strain is caused by the mecI-mediated repression of PBP 2' production. Antimicrob. Agents Chemother. 40: 2680-5685.

7. Holden MT et al. (2004) Complete genomes of two clinical Staphylococcus aureus strains: evidence for the rapid evolution of virulence and drug resistance. Proc Natl Acad Sci U S A 101: 9786–9791.

8. Charpentier E et al. (2004) Novel cassette-based shuttle vector system for gram-positive bacteria. Appl. Environ. Microbiol. 70: 6076-6085.

9. Brückner R (1997) Gene replacement in Staphylococcus carnosus and Staphylococcus xylosus. FEMS Microbiol Lett. 151: 1-8.
