# Supplementary material for: A Staphylococcus aureus Small RNA Is Required for Bacterial Virulence and Regulates the Expression of an Immune-Evasion Molecule
Source: PLoS Pathog. 2010 Jun 3;6(6):e1000927. doi: 10.1371/journal.ppat.1000927 (PMC2880579; doi:10.1371/journal.ppat.1000927)
Supplement: Table S3 — DNA primers used in this study. (0.08 MB DOC) [file ppat.1000927.s009.doc]

**Table S3.** DNA primers used in this study

| **DNAs** | **Sequences** | **Purposes** |
| --- | --- | --- |
| Anti1640 | GGCGCTccttgaaaacgccc | SprD northern/5’ RACE |
| Antisprd3 | GCCCGAAAAGGAGCAATACAT | *sprD* 5’ RACE |
| Sbineg5 | tcactcgcttttgcttcccca | *sbi* 5’ RACE  *sbi* 5’ RACE |
| Sbineg3 | tcgcgtaatgttttgatgtattgg |
| 5pstSBI | ttaaatCTGCAGagataatgcaatagtagccat | pCN38Ω*sprD*, pCN35Ω*sprD* |
| 3ecoriSBI | TAAATTGGAATTCCGACATACCTTATTGTATTTATAG |
| SprD5PstI | ttacgctgcagactattgcatttctccatata | pCN35Ω*sbi* |
| SprD3EcoRI | taacggaattcaaggattatttaacaagttgca |
| sprDforTR | TAATACGACTCACTATAGgggcgttttcaaggagcgcc | SprD  Transcription,  pCN38Ω*sprΔ*36 |
| sprDrevTR | AAGgtaagcaccgaaatgcttac |
| T7sprD_delfor | GCGCCTTTCATTTTTTATGTTTGCGCTTTCCAAATCAA |
| T7sprD_delre | TTGATTTGGAAAGCGCAAACATAAAAAATGAAAGGCGC |
| SBIforTR | TAATACGACTCACTATAGgcatacaataaatttaatatgtaa | Sbi mRNA transcription |
| SBIrevTR | gttgttttgagttgtttggtgct |
| SBIdeltaTR | TAATACGACTCACTATAGgCgaagttgctagttggggcagca |
| D1_1 | tcgctctagagaattcgtggacaacgtcgttatatagcg | *sprD* deletion |
| D1_2 | gcccggatccaaggtaatattaacataaaatggctac |
| D1_3 | gcaatagtagccattttatgttaatattaccttggatccgggcccacctaggaattgaat |
| D1_4 | atcaaattgacataccttattgtatttatagactcgagcgccgcggaaaactggt |
| D1_5 | gcggcgctcgagtctataaatacaataaggtatgtcaatttg |
| D1_6 | agcggagctcgaattcgaagggtgtgtattgttatttaccta |
| SBIDN315_1 | ttactgGAATTCtcaacattacaagaccactga | *sbi* deletion |
| SBIDN315_2 | gattgagaatcagcggttctactaattattaactaatgtt |
| SBIDN315_3 | aacattagttaataattagtagaaccgctgattctcaatc |
| SBIDN315_4 | ttacgAAGCTTGGATTCagatacttgaccattcggtg |
| RNAIIID_1 | ttacgAAGCTTGGATTCtggagatagttctaaaaatgaaac | *RNAIII* deletion |
| RNAIIID_2 | cttttagcatgttttaatataactgtaatgaagaagggatgagtt |
| RNAIIID_3 | aactcatcccttcttcattacagttatattaaaacatgctaaaag |
| RNAIIID_4 | ttactgGAATTCtaagctgcgatgttaccaatgt |
