# Supplementary material for: A Staphylococcus aureus Small RNA Is Required for Bacterial Virulence and Regulates the Expression of an Immune-Evasion Molecule
Source: PLoS Pathog. 2010 Jun 3;6(6):e1000927. doi: 10.1371/journal.ppat.1000927 (PMC2880579; doi:10.1371/journal.ppat.1000927)
Supplement: Supplementary file 5 [file ppat.1000927.s002.doc]

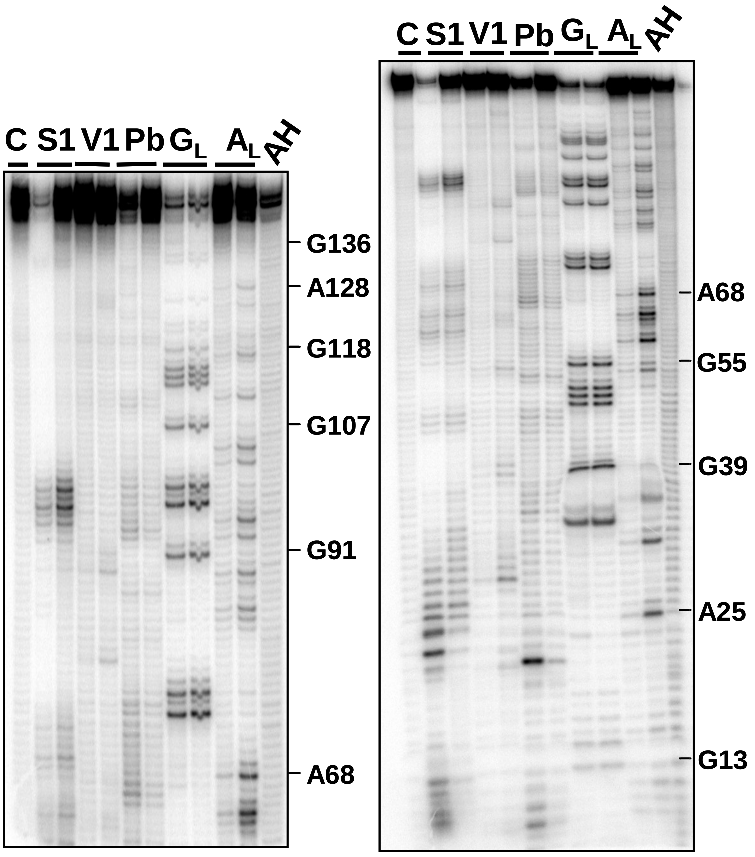

C
S1
V1
Pb
G_L
A_L
AH
G136
A128
G118
G107
G91
A68
C
S1
V1
Pb
G_L
A_L
AH
A68
G55
G39
A25
G13
