# Supplementary material for: A Staphylococcus aureus Small RNA Is Required for Bacterial Virulence and Regulates the Expression of an Immune-Evasion Molecule
Source: PLoS Pathog. 2010 Jun 3;6(6):e1000927. doi: 10.1371/journal.ppat.1000927 (PMC2880579; doi:10.1371/journal.ppat.1000927)
Supplement: Supplementary file 6 [file ppat.1000927.s003.doc]

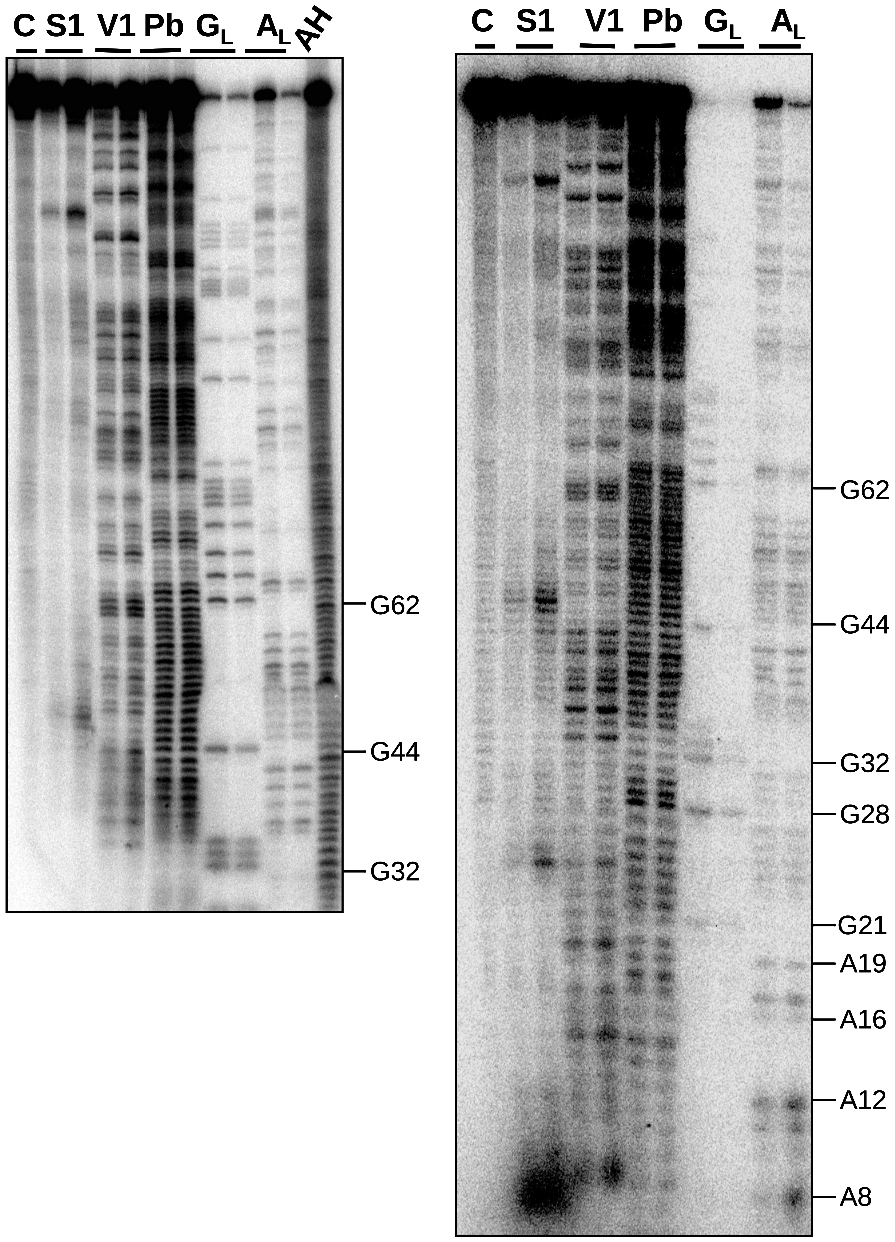
C S1 V1 Pb GL AL AH
G62
G44
G32
C S1 V1 Pb GL AL
G62
G44
G32
G28
G21
A19
A16
A12
A8
