# Supplementary material for: A Staphylococcus aureus Small RNA Is Required for Bacterial Virulence and Regulates the Expression of an Immune-Evasion Molecule
Source: PLoS Pathog. 2010 Jun 3;6(6):e1000927. doi: 10.1371/journal.ppat.1000927 (PMC2880579; doi:10.1371/journal.ppat.1000927)
Supplement: Supplementary file 7 [file ppat.1000927.s005.doc]

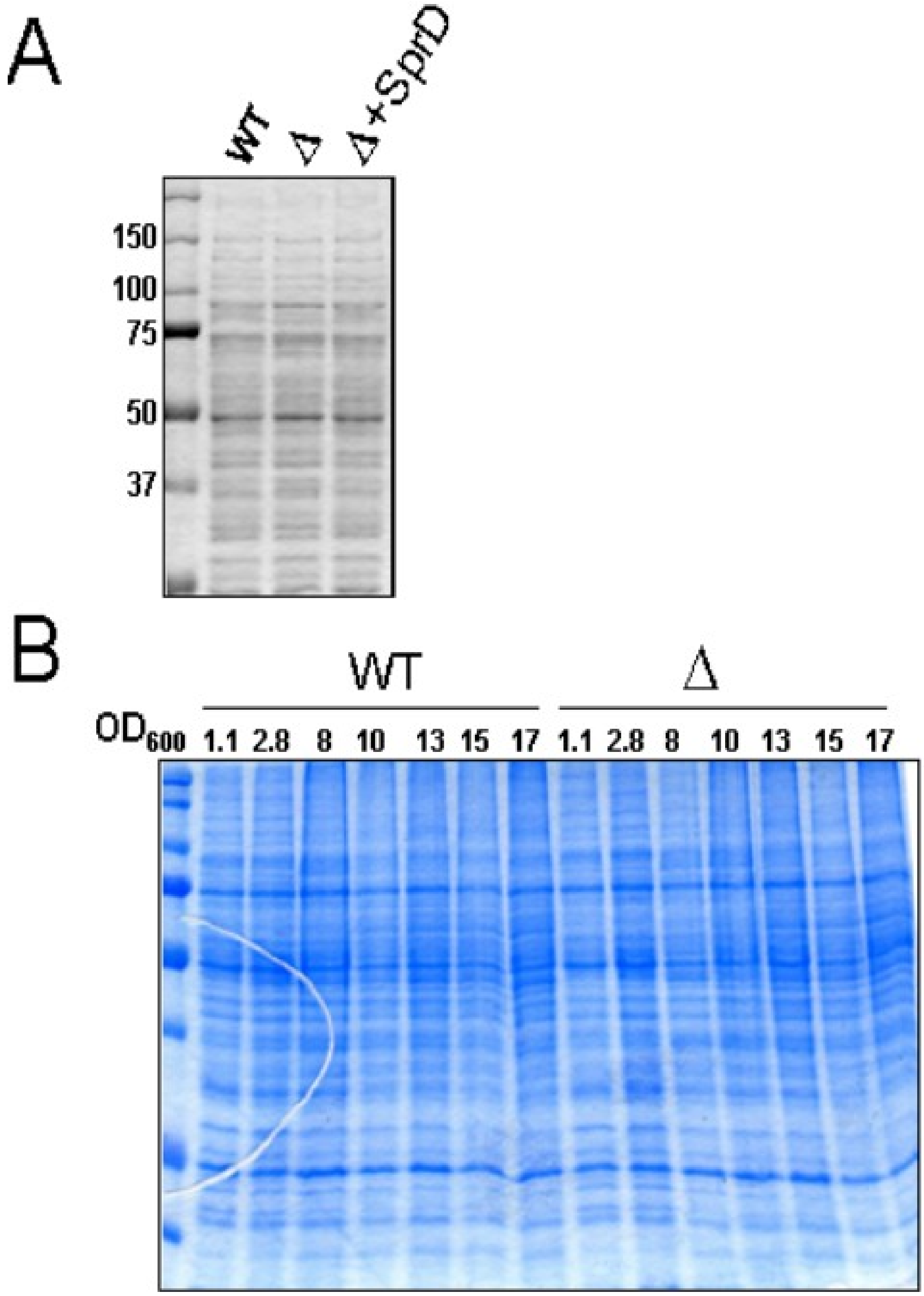
A
WT
Δ
Δ+SprD
150
100
75
50
37
B
WT
Δ
OD600
1.1
2.8
8
10
13
15
17
1.1
2.8
8
10
13
15
17
